# Clinical and Experimental Immunomodulation

**DOI:** 10.1155/2013/801251

**Published:** 2013-12-19

**Authors:** Lenin Pavón, Hugo Besedosky, Oscar Bottasso, Rogelio Hernández, Marco Velasco, Roger Loria

**Affiliations:** ^1^Department of Psychoimmunology, National Institute of Psychiatry “Ramón de la Fuente”, Calzada México-Xochimilco 101, Colonia San Lorenzo Huipulco, Tlalpan, 14370 Mexico City, DF, Mexico; ^2^Research Group Immunophysiology, Institute of Physiology and Pathophysiology, Philipps University, 35037 Marburg, Germany; ^3^Instituto de Inmunología, Facultad de Ciencias Médicas, Universidad Nacional de Rosario, Santa Fe 3100 CP, 2000 Rosario, Argentina; ^4^Department of Pathology, National Institute of Medical Sciences and Nutrition “Salvador Zubirán”, 14000 Mexico City, DF, Mexico; ^5^Department of Pharmacology, School of Medicine, National Autonomous University of Mexico, P.O. Box 70-297, Coyoacan, 04510 Mexico City, DF, Mexico; ^6^Department of Microbiology, Immunology, Virginia Commonwealth University, 1101 E. Marshal Street, Richmond, VA 232980678, USA

The inflammatory response is modulated by the concentration of soluble mediators and the coordinated action of different types of immune cells. Furthermore, basic and clinical research has demonstrated that the immune response is regulated by several factors such as: the chemical nature and the concentration of antigen; the route of administration; the cell type involved in the antigen presentation to their specific lymphocytes; and the presence of antibodies and/or immune complexes among other mechanisms.

More recently, it has been described that other signaling molecules like neurotransmitters and hormones can also modulate the immune response. Over time, this information has enabled the elucidation of the role of immune cell products in physiological processes like sleep, memory, learning, and pain, or in autoimmune and infective diseases, as well as the mechanisms involved. Such evidence provides the opportunity for the development of novel therapeutic approaches for diseases with deleterious immune and inflammatory components. The papers presented in this special issue focus on the leveraging knowledge of clinical and experimental immunomodulation.

First, the reader can find seven experimental approaches that analyze immunomodulation mediated by hormones, neurotransmitters, cytokines, and antigens. The work of M. V. Legorreta-Haquet et al. shows that prolactin in early stages of B cells maturation process may promote the survival of self-reactive clones in a murine model of lupus. T. Schaumann et al. present results of anti-inflammatory effects of glycine in gingival inflammation and encourage further research on the utility of glycine in the prevention therapy of inflammatory periodontitis. B. Dénes et al. share an interesting work on experimental immunotherapy with a multicomponent vaccine containing a cholera toxin B subunit-autoantigen fusion protein for restoration of euglycemia and immunological homeostasis in NOD mice. F. Robledo-Ávila et al. explored a novel therapeutic approach consisting in the administration of murine dialyzable leukocyte extracts plus a reduced, and therefore less toxic, dose of Amphotericin B in a mouse model of systemic candidiasis. The approach proved to be effective in reducing mortality, pathogen burden, and tissue damage at the renal level. S. Mburu et al. evaluated *in vitro* the modulation of LPS-induced CD4+ T cell activation and apoptosis by antioxidants in cells from untreated asymptomatic HIV infected participants. Their results set the basis for the development of an adjuvant therapy aimed to counteract the harmful effects of chronic immune activation on CD4+ T cells. S. Dang et al. show that LMW-HA modulates papillary thyroid carcinoma (PTC) cell behavior via TLR-4 signaling providing examples of the functional roles of CXCR7 in proliferation and migration. Their data are elegantly complemented with the analysis of TLR4 and CXCR7 expression in PTC clinical samples. Finally, J. M. Calleja-Castillo et al. investigate the effect of deep brain stimulation (DBS) at hypothalamic nucleus in Wistar rats, over the circulating concentrations of corticosterone and proinflammatory cytokines, detecting that the chronic application of this therapy to Wistar rats induces a significant circulatory rise in inflammatory mediators and blocks HPA axis activity. These results suggest that immunity might be altered in patients who are treated with DBS and provide the basis for the development of strategies to prevent immunity-related secondary effects of DBS.

Regarding the clinical approaches of immunomodulation, three works are also included. The first one, from N. Valero-Pacheco et al., analyzes the expression of PD-L1 on T cells in patients infected with the influenza virus A(H1N1)pdm09 and its impact on T cell responses. The second one, from J. Galicia-Carreón et al., studies the context of the unbalanced immunological mechanisms underlying the development of allergic conjunctivitis by evaluating the frequency of Tregs as well as cells expressing homing receptors in peripheral blood from patients. The third one, from M. E. Hernández et al., presents the results of a clinical followup of major depressive disorder (MDD) patients treated with a combination of selective serotonin reuptake inhibitors (SSRI) and human dialyzable leukocytes extract (hDLE) as immunomodulator. The latter consists of small weight peptides and has been used successfully as adjuvant therapy in diverse infectious and deficient cell-immunity problems. MDD patients present imbalances in neurotransmitter levels, hormones such as cortisol, and cytokines that contribute to the behavioral and immune disturbances observed in them. This combined treatment efficiently restored the pro- and anti-inflammatory cytokine balance and cortisol levels when compared with patients treated only with SSRI. This study constitutes the first report of a clinical assay that analyzes the effects of immunotherapy in MDD.

This special issue also includes two reports of experimental techniques that allow the assessment of immunomodulation. The work of I. Lima Siman et al. evaluated the serum levels of allergen-specific IgG antibodies from atopic patients. The authors conclude that this laboratory test would help specialists to follow up patients under immunotherapy. The report by M. C. Jiménez-Martínez et al. shows an experimental technique to identify NnTreg lymphocytes by staining them with *Amaranthus leucocarpus* lectin and posterior FACS.

Last but not least, this issue presents four revisions on a broad range of topics. N. Deckx et al. focus on multiple sclerosis and discuss the influence of neuroendocrine immune system over the susceptibility and severity of autoimmune diseases, as well as new therapeutic approaches for the treatment of this kind of diseases. R. Cabezón and D. Benitez-Ríbas review the participation of different dendritic cells (DCs) subsets and their role in inflammatory bowel disease and present preclinical studies performed in animal models describing the recent characterization of tol-DCs from Crohn's disease patients. G. A. Toledo-Ibarra et al. describe some aspects of the immunity of fish and its connections with cholinergic system, highlighting the possibility that bidirectional communication between the nervous and immune systems exists in lower vertebrates as well as during evolution of immune system. G. Hurtado-Alvarado et al. present an extensive review focused on the relationship between inflammation and inflammatory markers as well as sleep and sleep loss.

